# Racial/ethnic differences in time to nephrectomy and its association with cancer-specific mortality in localized renal cell carcinoma

**DOI:** 10.1007/s11255-026-05141-0

**Published:** 2026-04-16

**Authors:** Maximilian Filzmayer, Federico Polverino, Michele Petix, Leonardo Quarta, Filippo Orlandi, Jordan A. Goyal, Nicola Longo, Gennaro Musi, Alberto Briganti, Salvatore Micali, Shahrokh F. Shariat, Marina Kosiba, Clara Humke, Mike Wenzel, Fred Saad, Felix K.-H. Chun, Pierre I. Karakiewicz

**Affiliations:** 1 Cancer Prognostics and Health Outcomes Unit, Division of Urology, University of Montreal Health Center, 1000 Rue Saint-Denis, Montreal, H2X 0C1 Canada; 2 Department of Urology, Goethe University, University Hospital, Frankfurt am Main, Germany; 3 Department of Neurosciences, Science of Reproduction and Odontostomatology, University of Naples Federico II, Naples, Italy; 4 Università Degli Studi Di Milano, Milan, Italy; 5 Department of Urology, IEO European Institute of Oncology, IRCCS, Milan, Italy; 6 Division of Experimental Oncology/Unit of Urology, URI, Urological Research Institute, IRCCS San Raffaele Scientific Institute, Milan, Italy; 7 Vita-Salute San Raffaele University, Milan, Italy; 8 Department of Urology, AOU di Modena, University of Modena and Reggio Emilia, Modena, Italy; 9 Department of Urology, Comprehensive Cancer Center, Medical University of Vienna, Vienna, Austria; 10 Department of Urology, Weill Cornell Medical College, New York, NY USA; 11 Department of Urology, University of Texas Southwestern Medical Center, Dallas, TX USA; 12 Hourani Center of Applied Scientific Research, Al-Ahliyya Amman University, Amman, Jordan

**Keywords:** Kidney cancer, Nephrectomy, Time to surgical treatment, Race/ethnicity, Survival

## Abstract

**Purpose:**

Whether racial/ethnic differences exist in time to nephrectomy (TTN) and whether prolonged TTN differentially affects cancer-specific mortality (CSM) remains unclear. We evaluated race/ethnicity as a predictor of prolonged TTN and assessed race/ethnicity-specific associations between TTN and CSM in localized renal cell carcinoma (RCC).

**Methods:**

Patients were identified within the Surveillance, Epidemiology and End Results database (2010–2021) and stratified according to race/ethnicity and TTN ≤ 3 vs. > 3 months. Multivariable logistic regression models, propensity score matching (PSM) and multivariable competing risks regression models were used.

**Results:**

In 11,058 T1b–2 N0 M0 clear-cell RCC patients, TTN > 3 months was recorded in 1168 (15.6%) of 7506 Caucasians, in 505 (23.6%) of 2138 Hispanics, in 180 (26.6%) of 676 African Americans, and in 118 (16.0%) of 738 Asians and Pacific Islanders (API). Hispanic (OR 1.80, p < 0.001) and African American (OR 2.10, p < 0.001) race/ethnicity independently predicted higher proportions of TTN > 3 months, compared to Caucasian. Over the study span, the proportion of patients with TTN > 3 months increased significantly in all four racial/ethnic groups (all p < 0.01). After PSM, TTN > 3 months was associated with higher CSM in Caucasians (sHR 1.57, p < 0.001) and in Hispanics (sHR 1.55, p = 0.046), but not in African Americans or APIs.

**Conclusion:**

In localized RCC patients treated with nephrectomy, TTN > 3 months became more prevalent over time in all four examined racial/ethnic groups. In Hispanics and African Americans, TTN > 3 months proportions were higher than in Caucasians and APIs. TTN > 3 months was independently associated with higher CSM in Caucasians and Hispanics, but not in African Americans and APIs.

**Supplementary Information:**

The online version contains supplementary material available at 10.1007/s11255-026-05141-0.

## Background

Longer time interval between initial diagnosis and nephrectomy (TTN) was identified as determinant of worse overall mortality in renal cell carcinoma (RCC) [1–7]. However, no previous study stratified its findings according to race/ethnicity. Racial/ethnic disparities in RCC incidence, management, and cancer-control outcomes have been repeatedly documented, but these studies did not account for TTN as a potential contributing factor [8–15]. Several investigations addressed predictors of prolonged TTN, showing that non-Caucasian patients were more likely to experience surgical delay in high-stage RCC and other solid tumors [1–3, 16]. However, no study has tested whether prolonged TTN differentially affects oncologic outcomes within specific racial/ethnic groups.

Therefore, it remains unknown whether TTN differs between racial/ethnic groups in localized RCC and whether the adverse effect of prolonged TTN on cancer-specific mortality (CSM) varies by race/ethnicity. We hypothesized that rates of prolonged TTN as well as its effect on CSM may depend on race/ethnicity. We addressed this knowledge gap and tested these hypotheses within a large population-based database (Surveillance, Epidemiology and End Results [SEER] 2010–2021) [17].

## Methods

### Data source and study population

Within the SEER database (2010–2021), we identified adult patients with histologically confirmed T1b–2 RCC (International Classification of Diseases for Oncology site code C64.9) treated with either partial or radical nephrectomy [17]. T1a tumors were not included since active surveillance or local tumor destruction may be recommended in many such patients [18–20]. Conversely, nephrectomy, either partial or radical, represents the standard of care for T1b, T2a, and T2b tumors [18–20]. Patients with non-clear-cell histology or exceptional tumor sizes in excess of the 99th percentile were also not considered in the analyses to ensure cohort homogeneity [21–23]. Further exclusions were missing information regarding TTN, follow-up, or patient and tumor characteristics. TTN was defined as the interval between the date of diagnosis and the date of surgery as reported within SEER. Because the SEER date of diagnosis may reflect the date of registry entry rather than the first clinical suspicion, some degree of misclassification of TTN cannot be excluded. Race/ethnicity was used as reported within SEER, following established SEER coding conventions [17]. The cohort consisted of the four predominant racial/ethnic groups represented in SEER: Caucasians, Hispanics, African Americans, and Asians and Pacific Islanders (API). Patients were further stratified according to TTN ≤ 3 vs. > 3 months. This threshold was used in several previously reported analyses of prolonged TTN in RCC [3–6].

### Outcomes of interest

The primary outcome of interest was CSM, defined as death attributable to RCC as recorded within the SEER database [17]. Other-cause mortality (OCM) was defined as death from non-RCC-related causes and was treated as a competing risk event. CSM rates were estimated at 120 months from the date of surgery. Secondary outcomes included predictor status of race/ethnicity for TTN > 3 months and temporal trends in proportions of TTN > 3 months.

### Statistical analyses

First, a multivariable logistic regression model (mLRM) addressed the association between TTN > 3 months and race/ethnicity. Covariates were year of diagnosis (continuous), age (continuous), sex (male vs. female), income (low [annual income below the cohort median of 80,000 USD] vs. high), area of residence (urban vs. rural), tumor size (continuous), and nephrectomy type (radical vs. partial). Subsequent analyses were conducted separately for each racial/ethnic group (Caucasians, Hispanics, African Americans, and APIs). Second, the estimated annual percentage change (EAPC) of the proportion of patients with TTN > 3 months were calculated using log-linear regression. Third, one-to-two propensity score matching (PSM) according to the nearest neighbor method without replacement and without caliper was applied between patients with TTN > 3 vs. ≤ 3 months. PSM relied on year of diagnosis, age, sex, income, tumor size, T substage (T1b vs. T2a vs. T2b), nephrectomy type, and tumor grade group (G1–2 vs. G3–4). The aim of PSM was to maximally reduce or ideally eliminate differences between patients with TTN > 3 vs. ≤ 3 months that may represent bias or confounders. Fourth, cumulative incidence plots depicted 120-month CSM rate differences between patients with TTN > 3 vs. ≤ 3 months. Fifth, multivariable Fine-Gray competing risks regression (CRR) models addressing CSM were fitted to test the effect of TTN > 3 months [24]. Covariates were year of diagnosis, age, sex, income, area of residence, tumor size, nephrectomy type, and tumor grade group.

Sixth, sensitivity analyses were performed using alternative TTN cutoffs of > 2 and > 4 months. All analytical steps described above were repeated accordingly. In addition, formal interaction testing between TTN > 3 months and race/ethnicity was performed within a unified CRR model after PSM of the overall cohort.

R software was used for statistical computing and graphics (R version 4.4.3, The R Foundation for Statistical Computing, Vienna, Austria). All tests were two-sided with significance set at p < 0.05. All reported odds ratios (OR) and subdistribution hazard ratios (sHR) derived from multivariable adjusted regression models.

## Results

### Proportions of TTN > 3 months according to race/ethnicity

Within the SEER database (2010–2021) we identified 11,058 T1b–2 N0 M0 RCC patients treated with radical or partial nephrectomy (Table 1). Of those, 7506 (67.9%) were Caucasians, 2138 (19.3%) were Hispanics, 676 (6.1%) were African Americans, and 738 (6.7%) were APIs. TTN > 3 months was recorded in 1168 (15.6%) Caucasians, in 505 (23.6%) Hispanics, in 180 (26.6%) African Americans, and in 118 (16.0%) APIs.

**Table 1:** Descriptive characteristics of 11,058 T1b–2 N0 M0 clear-cell renal cell carcinoma patients treated with partial or radical nephrectomy within the SEER database (2010–2021)

Characteristics	Overall, n = 11,058
Time to nephrectomy	
Median (IQR) in days	44 (25, 75)
> 3 months, n (%)	1971 (17.8%)
Race/ethnicity, n (%)	
Caucasians	7506 (67.9%)
Hispanics	2138 (19.3%)
African Americans	676 (6.1%)
Asians and Pacific Islanders	738 (6.7%)
Year of diagnosis, median (IQR)	2016 (2014, 2019)
Age in years, median (IQR)	62 (54, 70)
Female sex, n (%)	4022 (36.4%)
Low income^a^, n (%)	5819 (52.6%)
Urban residence, n (%)	9513 (86.0%)
Tumor size in cm, median (IQR)	5.6 (4.8, 7.2)
T substage, n (%)	
T1b	8099 (73.2%)
T2a	2288 (20.7%)
T2b	671 (6.1%)
Radical Nephrectomy, n (%)	8105 (73.3%)
Tumor grade group G3–4, n (%)	4297 (38.9%)

^a^defined by annual income below cohort median (80,000 USD)

After multivariable adjustment in mLRM, Hispanics (OR 1.80, 95% confidence interval [CI] 1.60–2.04, p < 0.001) and African Americans (OR 2.10, 95% CI 1.74–2.53, p < 0.001) exhibited significantly higher odds of TTN > 3 months compared with Caucasians (Table 2). No independent association was observed for APIs (p = 0.5).

**Table 2:** Multivariable logistic regression model predicting time to nephrectomy > 3 vs. ≤ 3 months according to race/ethnicity in 11,058 T1b–2 N0 M0 clear-cell renal cell carcinoma patients treated with partial or radical nephrectomy within the SEER database (2010–2021)

Race/ethnicity	TTN ≤ 3 months, n (%)	TTN > 3 months, n (%)	mLRM predicting TTN > 3 months (vs. ≤ 3 months)^a^		
			Odds ratio	95% confidence interval	p-value
Caucasians	6338 (84.4%)	1168 (15.6%)	Ref		
Hispanics	1633 (76.4%)	505 (23.6%)	1.80	1.60–2.04	< 0.001
African Americans	496 (73.4%)	180 (26.6%)	2.10	1.74–2.53	< 0.001
Asians and Pacific Islanders	620 (84.0%)	118 (16.0%)	1.08	0.87–1.33	0.5

^a^adjusted for year of diagnosis, age, sex, income, area of residence, tumor size, and nephrectomy type

**Fig. 1:**
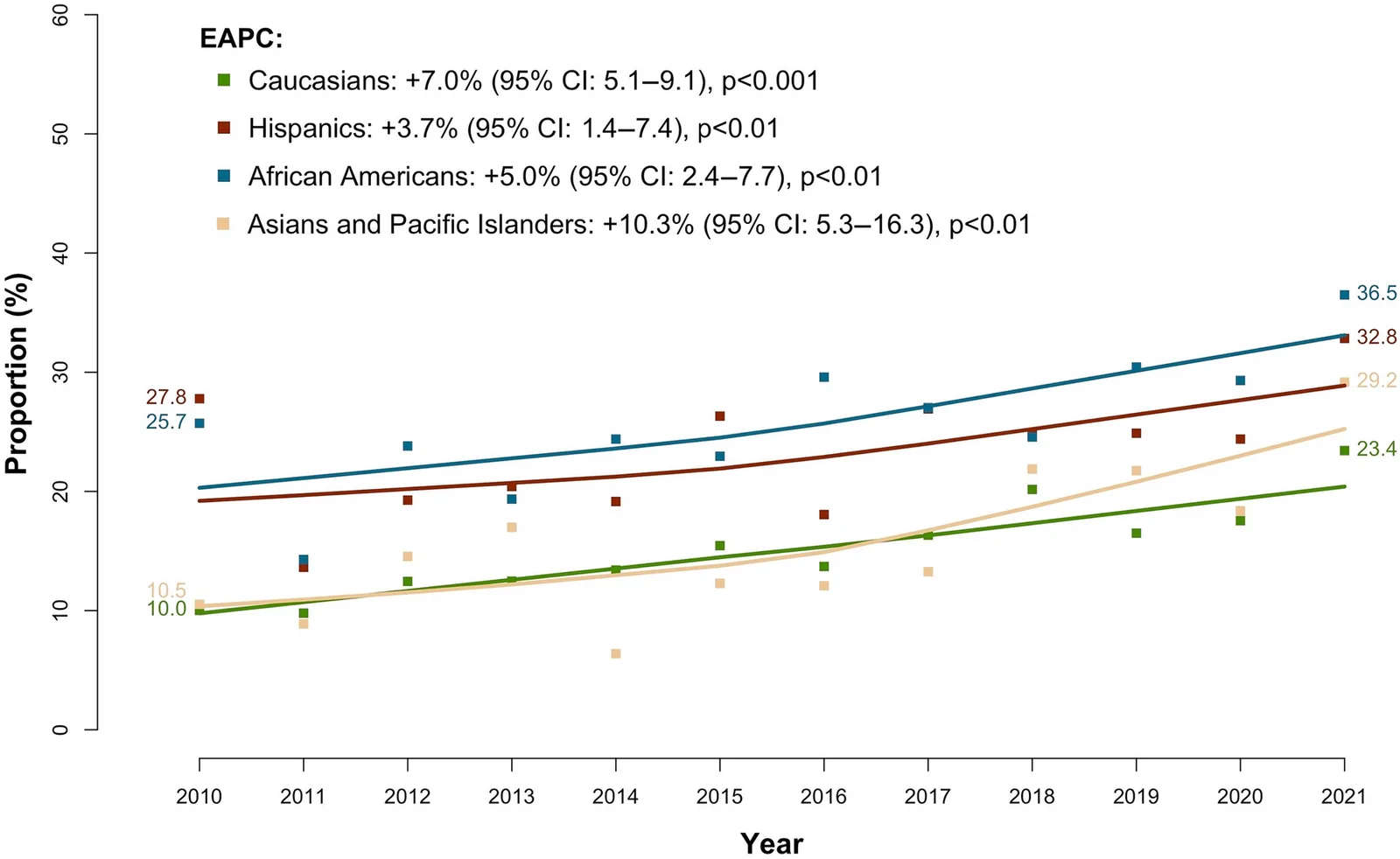
Proportions of patients with time to nephrectomy > 3 months over time among 11,058 T1b–2 N0 M0 clear-cell renal cell carcinoma patients treated with partial or radical nephrectomy within the SEER database (2010–2021), stratified according to race/ethnicity, with estimated annual percentage change. *CI* confidence interval, *EAPC* Estimated annual percentage change, *SEER* Surveillance, epidemiology, and end results, *TTN* Time to nephrectomy

Over time, the proportion of patients with TTN > 3 months increased significantly in all four examined racial/ethnic groups (all p < 0.01; Fig. 1). Specifically, in Caucasians, the proportion of TTN > 3 months increased from 10.0% in 2010 to 23.4% in 2021 (EAPC + 7.0%). In Hispanics, the proportion increased from 27.8 to 32.8% (EAPC + 3.7%). In African Americans, the proportion increased from 25.7 to 29.3% (EAPC + 5.0%), and in APIs, the proportion increased from 10.5 to 23.4% (EAPC + 10.3%).

### Propensity score matching

Before PSM, significant differences between patients with TTN > 3 months and those with TTN ≤ 3 months were observed across all four racial/ethnic groups (Tables 3, 4, 5 and 6). Specifically, patients with TTN > 3 months were treated in more recent years, were older, harbored smaller tumor sizes and lower T substages, and underwent radical nephrectomy less frequently than those with TTN ≤ 3 months. After PSM, no significant residual differences in baseline characteristics remained between patients with TTN ≤ 3 vs. > 3 months within each racial/ethnic group.

**Table 3:** Descriptive characteristics of 7506 Caucasian T1b–2 N0 M0 clear-cell renal cell carcinoma patients treated with partial or radical nephrectomy within the SEER database (2010–2021), stratified by time to nephrectomy ≤ 3 vs. > 3 months, before and after 1:2 propensity score matching

Caucasians							
Characteristics	Before PSM				After PSM^a^		
	Overall, n = 7506	TTN ≤ 3 months n = 6338 (84.4%)	TTN > 3 months n = 1168 (15.6%)	p-value^b^	TTN ≤ 3 months n = 2336 (36.9%)	TTN > 3 months n = 1168 (100%)	p-value^b^
Year of diagnosis, median (IQR)	2016 (2014, 2019)	2016 (2013, 2019)	2017 (2015, 2019)	< 0.001	2017 (2015, 2019)	2017 (2015, 2019)	0.7
Age in years, median (IQR)	63 (55, 71)	63 (55, 70)	65 (57, 73)	< 0.001	66 (58, 73)	65 (57, 73)	0.9
Female sex, n (%)	2602 (34.7%)	2238 (35.3%)	364 (31.2%)	0.006	730 (31.3%)	364 (31.2%)	> 0.9
Low income^c^, n (%)	4180 (55.7%)	3544 (55.9%)	636 (54.5%)	0.4	1249 (53.5%)	636 (54.5%)	0.6
Urban residence, n (%)	6124 (81.6%)	5192 (81.9%)	932 (79.8%)	0.1	1930 (82.6%)	932 (79.8%)	0.1
Tumor size in cm, median (IQR)	5.6 (4.8, 7.2)	5.8 (4.9, 7.5)	5.3 (4.5, 6.3)	< 0.001	5.2 (4.5, 6.3)	5.3 (4.5, 6.3)	0.6
T substage, n (%)				< 0.001			> 0.9
T1b	5517 (73.5%)	4534 (71.5%)	983 (84.2%)		1964 (84.1%)	983 (84.2%)	
T2a	1,529 (20.4%)	1373 (21.7%)	156 (13.4%)		313 (13.4%)	156 (13.4%)	
T2b	460 (6.1%)	431 (6.8%)	29 (2.5%)		59 (2.5%)	29 (2.5%)	
Radical Nephrectomy, n (%)	5514 (73.5%)	4787 (75.5%)	727 (62.2%)	< 0.001	1481 (63.4%)	727 (62.2%)	0.5
Tumor grade group G3–4, n (%)	2917 (38.9%)	2482 (39.2%)	435 (37.2%)	0.2	884 (37.8%)	435 (37.2%)	0.7

^a^PSM relied on year of diagnosis, age, sex, income, tumor size, T substage, nephrectomy type, and tumor grade group

^b^Wilcoxon rank sum test, Pearson’s chi-squared test; after PSM all covariates were well balanced (standardized mean differences < 0.1); covariate balance is illustrated in Supplementary Fig. 1

^c^defined by annual income below cohort median (80,000 USD)

### Effect of TTN > 3 months on CSM

**Fig. 2:**
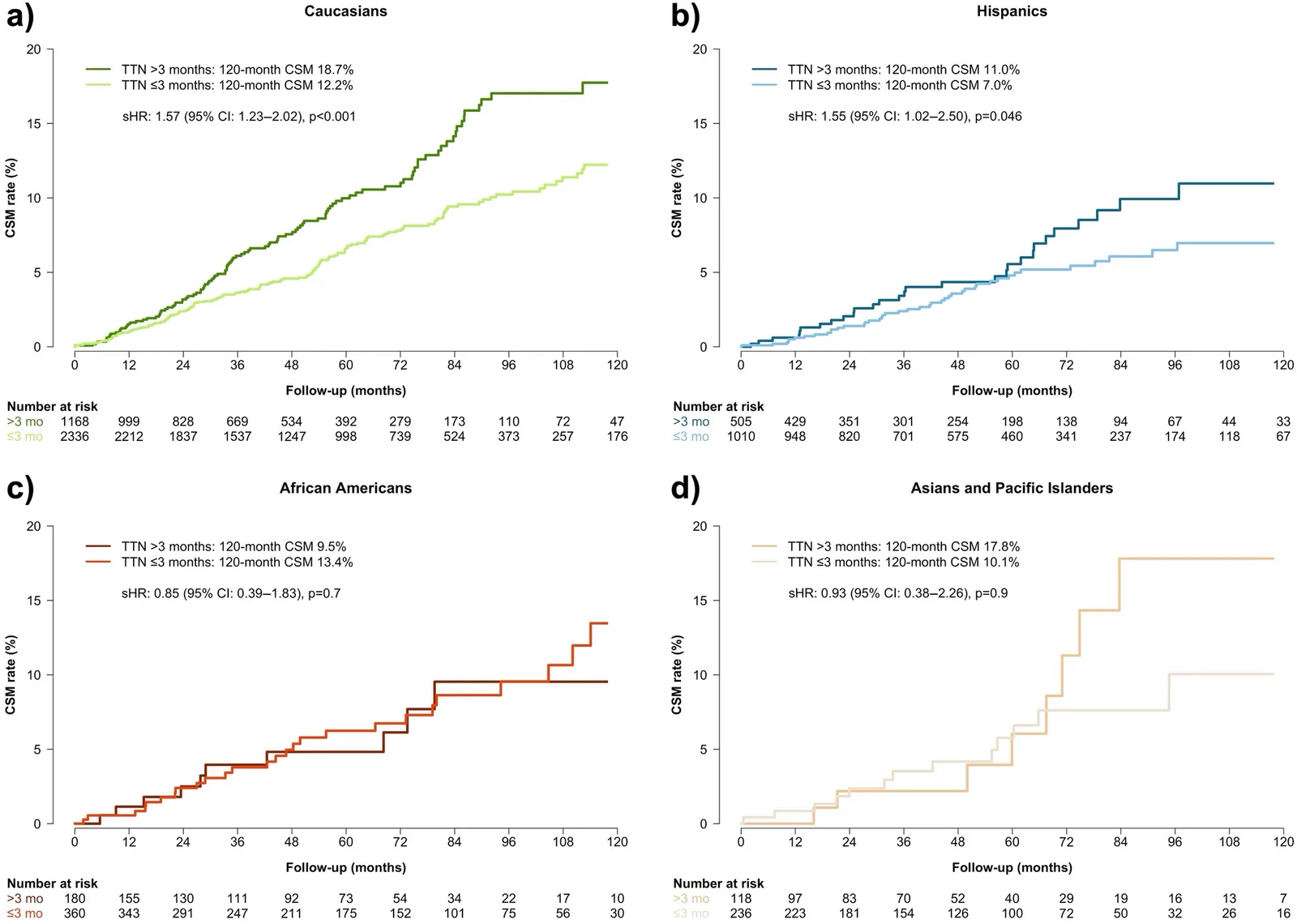
Cumulative incidence plots depicting cancer-specific mortality of Caucasians **a**, Hispanics **b**, African Americans **c**, and Asians or Pacific Islanders **d** with T1b–2 N0 M0 clear-cell renal cell carcinoma treated with partial or radical nephrectomy within the SEER database (2010–2021). Patients were stratified according to time to nephrectomy > 3 months vs. ≤ 3 months and matched 1:2 using propensity score matching. Subdistribution hazard ratios derived from multivariable competing risks regression models adjusted for year of diagnosis, age, sex, income, area of residence, tumor size, nephrectomy type, and tumor grade group. *CI* Confidence interval, *CSM* Cancer-specific mortality, *sHR* Subdistribution hazard ratio, *SEER* Surveillance, epidemiology, and end results, *TTN* Time to nephrectomy

In Caucasians, all 1168 (100%) patients with TTN > 3 months were matched to 2336 (36.9%) of 6338 patients with TTN ≤ 3 months. At 120 months after nephrectomy, CSM rates were 18.7 vs. 12.2% in patients with TTN > 3 vs. ≤ 3 months (Fig. 2a). In multivariable CRR models addressing CSM after adjustment for OCM and covariates, TTN > 3 months was associated with a 1.57-fold higher CSM (95% CI 1.23–2.02, p < 0.001).

**Table 4:** Descriptive characteristics of 2138 Hispanic T1b–2 N0 M0 clear-cell renal cell carcinoma patients treated with partial or radical nephrectomy within the SEER database (2010–2021), stratified by time to nephrectomy ≤ 3 vs. > 3 months, before and after 1:2 propensity score matching

Hispanics							
Characteristics	Before PSM				After PSM^a^		
	Overall n = 2138	TTN ≤ 3 months n = 1633 (76.4%)	TTN > 3 months n = 505 (23.6%)	p-value^b^	TTN ≤ 3 months n = 1010 (61.8%)	TTN > 3 months n = 505 (100%)	p-value^b^
Year of diagnosis, median (IQR)	2017 (2014, 2019)	2016 (2014, 2019)	2017 (2015, 2019)	< 0.001	2017 (2015, 2019)	2017 (2015, 2019)	0.7
Age in years, median (IQR)	59 (50, 67)	58 (49, 67)	61 (52, 70)	< 0.001	60 (51, 69)	61 (52, 70)	0.6
Female sex, n (%)	866 (40.5%)	652 (39.9%)	214 (42.4%)	0.3	425 (42.1%)	214 (42.4%)	> 0.9
Low income^c^, n (%)	1005 (47.0%)	773 (47.3%)	232 (45.9%)	0.4	474 (46.9%)	232 (45.9%)	0.7
Urban residence, n (%)	2066 (96.6%)	1578 (96.6%)	488 (96.6%)	> 0.9	971 (96.1%)	488 (96.6%)	0.7
Tumor size in cm, median (IQR)	5.5 (4.7, 7.2)	5.8 (4.8, 7.5)	5.4 (4.5, 6.5)	< 0.001	5.3 (4.5, 6.5)	5.4 (4.5, 6.5)	0.9
T substage, n (%)				< 0.001			0.8
T1b	1568 (73.3%)	1160 (71.0%)	408 (80.8%)		823 (81.5%)	408 (80.8%)	
T2a	454 (21.2%)	377 (23.1%)	77 (15.2%)		142 (14.1%)	77 (15.2%)	
T2b	116 (5.4%)	96 (5.9%)	20 (4.0%)		45 (4.5%)	20 (4.0%)	
Radical Nephrectomy, n (%)	1546 (72.3%)	1209 (74.0%)	337 (66.7%)	< 0.001	672 (66.5%)	337 (66.7%)	> 0.9
Tumor grade group G3–4, n (%)	792 (37.0%)	622 (38.1%)	170 (33.7%)	0.1	340 (33.7%)	170 (33.7%)	> 0.9

^a^PSM relied on year of diagnosis, age, sex, income, tumor size, T substage, nephrectomy type, and tumor grade group

^b^Wilcoxon rank sum test, Pearson’s chi-squared test; after PSM all covariates were well balanced (standardized mean differences < 0.1); covariate balance is illustrated in Supplementary Fig. 1

^c^defined by annual income below cohort median (80,000 USD)

In Hispanics, all 505 (100%) patients with TTN > 3 months were matched to 1010 (61.8%) of 1633 patients with TTN ≤ 3 months. At 120 months after nephrectomy, CSM rates were 11.0 vs. 7.0% in patients with TTN > 3 vs. ≤ 3 months (Fig. 2b). In multivariable CRR models addressing CSM after adjustment for OCM and covariates, TTN > 3 months was associated with a 1.55-fold higher CSM (95% CI 1.02–2.50, p = 0.046).

In African Americans, all 180 (100%) patients with TTN > 3 months were matched to 360 (72.6%) of 496 patients with TTN ≤ 3 months. No 120-month CSM differences between TTN > 3 vs. ≤ 3 months were observed (Fig. 2c), and TTN > 3 months failed to achieve independent predictor status in multivariable CRR models addressing CSM (sHR 0.85, 95% CI 0.39–1.83, p = 0.7).

**Table 5:** Descriptive characteristics of 676 African American T1b–2 N0 M0 clear-cell renal cell carcinoma patients treated with partial or radical nephrectomy within the SEER database (2010–2021), stratified by time to nephrectomy ≤ 3 vs. > 3 months, before and after 1:2 propensity score matching

African Americans							
Characteristics	Before PSM				After PSM^a^		
	Overall, n = 676	TTN ≤ 3 months, n = 496 (73.4%)	TTN > 3 months, n = 180 (26.6%)	p-value^b^	TTN ≤ 3 months, n = 360 (72.6%)	TTN > 3 months, n = 180 (100%)	p-value^b^
Year of diagnosis, median (IQR)	2016 (2014, 2019)	2016 (2013, 2018)	2017 (2015, 2019)	0.016	2016 (2014, 2019)	2017 (2015, 2019)	0.2
Age in years, median (IQR)	62 (54, 70)	62 (54, 71)	61 (53, 68)	0.2	61 (54, 70)	61 (53, 68)	0.5
Female sex, n (%)	301 (44.5%)	224 (45.2%)	77 (42.8%)	0.6	158 (43.9%)	77 (42.8%)	0.8
Low income^c^, n (%)	443 (65.5%)	338 (68.1%)	105 (58.3%)	0.018	238 (66.1%)	105 (58.3%)	0.1
Urban residence, n (%)	620 (91.7%)	453 (91.3%)	167 (92.8%)	0.6	330 (91.7%)	167 (92.8%)	0.7
Tumor size in cm, median (IQR)	5.7 (4.8, 7.5)	6.0 (5.0, 7.8)	5.2 (4.5, 6.2)	< 0.001	5.5 (4.7, 6.5)	5.2 (4.5, 6.2)	0.2
T substage, n (%)				< 0.001			> 0.9
T1b	490 (72.5%)	331 (66.7%)	159 (88.3%)		312 (86.7%)	159 (88.3%)	
T2a	141 (20.9%)	123 (24.8%)	18 (10.0%)		41 (11.4%)	18 (10.0%)	
T2b	45 (6.7%)	42 (8.5%)	3 (1.7%)		7 (1.9%)	3 (1.7%)	
Radical Nephrectomy, n (%)	504 (74.6%)	396 (79.8%)	108 (60.0%)	< 0.001	239 (66.4%)	108 (60.0%)	0.1
Tumor grade group G3–4, n (%)	267 (39.5%)	204 (41.1%)	63 (35.0%)	0.2	122 (33.9%)	63 (35.0%)	0.8

^a^PSM relied on year of diagnosis, age, sex, income, tumor size, T substage, nephrectomy type, and tumor grade group

^b^Wilcoxon rank sum test, Pearson’s chi-squared test; after PSM all covariates were well balanced (standardized mean differences < 0.1); covariate balance is illustrated in Supplementary Fig. 1

^c^defined by annual income below cohort median (80,000 USD)

In APIs, all 118 (100%) patients with TTN > 3 months were matched to 236 (38.1%) of 620 patients with TTN ≤ 3 months. No 120-month CSM differences between TTN > 3 vs. ≤ 3 months were observed (Fig. 2d), and TTN > 3 months failed to achieve independent predictor status in multivariable CRR models addressing CSM (sHR 0.93, 95% CI 0.38–2.26, p = 0.9).

### Sensitivity analyses

Using alternative TTN cutoffs of > 2 and > 4 months, mLRM for delayed nephrectomy yielded consistent findings, with persistently higher odds among Hispanics and African Americans, whereas APIs remained not independently associated (Supplementary Tables 1 and 2). In corresponding stratified CRR analyses after PSM, prolonged TTN remained associated with higher CSM in Caucasians and partially in Hispanics, while no statistically significant associations were observed for African Americans or APIs (Supplementary Tables 3 and 4). Moreover, formal interaction testing between TTN > 3 months and race/ethnicity showed interaction estimates close to unity for Hispanics and below unity for African Americans and APIs compared with Caucasians, without reaching statistical significance (Supplementary Table 5).

**Table 6:** Descriptive characteristics of 738 Asian or Pacific Islandic T1b–2 N0 M0 clear-cell renal cell carcinoma patients treated with partial or radical nephrectomy within the SEER database (2010–2021), stratified by time to nephrectomy ≤ 3 vs. > 3 months, before and after 1:2 propensity score matching

Asians and Pacific Islanders							
Characteristics	Before PSM				After PSM^a^		
	Overall, n = 738	TTN ≤ 3 months, n = 620 (84.0%)	TTN > 3 months, n = 118 (16.0%)	p-value^b^	TTN ≤ 3 months, n = 236 (38.1%)	TTN > 3 months, n = 118 (100%)	p-value^b^
Year of diagnosis, median (IQR)	2016 (2013, 2019)	2016 (2013, 2018)	2018 (2015, 2020)	< 0.001	2017 (2015, 2019)	2018 (2015, 2020)	0.6
Age in years, median (IQR)	62 (53, 70)	62 (53, 70)	64 (53, 72)	0.003	64 (56, 71)	64 (53, 72)	0.8
Female sex, n (%)	253 (34.3%)	216 (34.8%)	37 (31.4%)	0.5	74 (31.4%)	37 (31.4%)	> 0.9
Low income^c^, n (%)	191 (25.9%)	161 (26.0%)	30 (25.4%)	> 0.9	60 (25.4%)	30 (25.4%)	> 0.9
Urban residence, n (%)	703 (95.3%)	591 (95.3%)	112 (94.9%)	0.8	223 (94.5%)	112 (94.9%)	> 0.9
Tumor size in cm, median (IQR)	5.6 (4.8, 7.5)	6.0 (5.0, 7.5)	5.1 (4.5, 6.0)	< 0.001	5.0 (4.5, 6.0)	5.1 (4.5, 6.0)	0.6
T substage, n (%)				< 0.001			0.9
T1b	524 (71.0%)	422 (68.1%)	102 (86.4%)		205 (86.9%)	102 (86.4%)	
T2a	164 (22.2%)	152 (24.5%)	12 (10.2%)		25 (10.6%)	12 (10.2%)	
T2b	50 (6.8%)	46 (7.4%)	4 (3.4%)		6 (2.5%)	4 (3.4%)	
Radical Nephrectomy, n (%)	541 (73.3%)	470 (75.8%)	71 (60.2%)	< 0.001	144 (61.0%)	71 (60.2%)	0.9
Tumor grade group G3–4, n (%)	321 (43.5%)	275 (44.4%)	46 (39.0%)	0.3	93 (39.4%)	46 (39.0%)	> 0.9

^a^PSM relied on year of diagnosis, age, sex, income, tumor size, T substage, nephrectomy type, and tumor grade group

^b^Wilcoxon rank sum test, Pearson’s chi-squared test; after PSM all covariates were well balanced (standardized mean differences < 0.1); covariate balance is illustrated in Supplementary Fig. 1

^c^defined by annual income below cohort median (80,000 USD)

## Discussion

In previously reported studies, TTN > 3 months adversely affects mortality in localized RCC [3, 4]. However, it remains unknown whether TTN differs between racial/ethnic groups in localized RCC and whether the adverse effect of TTN > 3 months on CSM varies by race/ethnicity. We addressed these knowledge gaps within the SEER database (2010–2021) and made several noteworthy observations.

First, we relied on a large, contemporary, population-based cohort of 11,058 patients with T1b–2 N0 M0 clear-cell RCC who predominantly underwent radical, but also partial, nephrectomy. Other studies that examined the effect of TTN on CSM mostly relied on substantially smaller sample sizes. For example, Mano et al. and Qi et al. [3, 5, 6]. examined single-center populations of 1484 and 561 patients, respectively, and Shiff et al. relied on 1769 patients within the Canadian Kidney Cancer Information System database Rosenblad et al. assessed 9918 patients within the National Swedish Kidney Cancer Register, whereas Srivastava et al. and Ginsburg et al. relied on the National Cancer Database, identifying 29,746 and 11,848 patients, respectively [2, 4, 7]. Although these latter three studies used comparably large datasets, their survival analyses exclusively relied on overall mortality. Since OCM contributes to overall mortality at a disproportionately higher level than CSM in localized RCC, databases lacking CSM information are truly not applicable to localized RCC patients, for whom CSM represents the essential metric of choice [25, 26]. Moreover, all six prior studies included all histological subtypes, unlike the current investigation, which focused exclusively on clear-cell RCC. Inclusion of non-clear-cell histological subtypes may dilute or obscure TTN-related effects on CSM, given their substantially less aggressive natural history [22, 23]. Most importantly, none of the prior investigations used race/ethnicity as a stratification variable in TTN-dependent survival analyses. This represents the key limitation of all prior studies and the central rationale for the current investigation.

Second, we observed that Hispanic (23.6%, OR 1.80) and African American (26.6%, OR 2.10) patients with localized RCC were significantly more likely to experience TTN > 3 months compared to Caucasians (15.6%), even after adjustment for socioeconomic characteristics, tumor size, and nephrectomy type. Conversely, APIs did not differ from Caucasians with respect to the proportion of TTN > 3 months (16.0%). These observations remained consistent in sensitivity analyses using alternative TTN cutoffs of > 2 and > 4 months. These findings can only be indirectly compared with a single previous investigation conducted in a different disease setting. In that study, Zeng et al. reported similarly elevated proportions of delayed nephrectomy among African American (OR 1.54) and Hispanic (OR 1.44) patients with locally advanced RCC, relative to Caucasians [1]. Since their observations cannot be generalized to patients with localized RCC, the current analysis represents the first evidence that race/ethnicity independently predicts delayed surgical treatment in such patients. This validates the rationale for the second part of the study, in which CSM is addressed according to TTN in a race/ethnicity-specific fashion.

Third, we observed a significant temporal increase in the proportion of patients experiencing TTN > 3 months across all four examined racial/ethnic groups. In 2010, the proportions of patients with TTN > 3 months were relatively low in Caucasians (10.0%) and APIs (10.5%), and markedly higher in Hispanics (27.8%) and African Americans (25.7%). However, consistent and statistically significant increases over time were documented in each group, resulting in 2021 proportions of 23.4% in Caucasians (EAPC + 7.0%), 32.8% in Hispanics (EAPC + 3.7%), 29.3% in African Americans (EAPC + 5.0%), and 23.4% in APIs (EAPC + 10.3%). To the best of our knowledge, this study represents the first to document race/ethnicity-specific temporal trends in surgical delay within localized RCC. Therefore, direct comparisons with existing literature cannot be made. The current findings underscore that prolonged TTN has become increasingly prevalent across all racial/ethnic groups and highlight the relevance of evaluating its potential prognostic implications. Potential explanations for this increase include rising case volumes, referral patterns, and healthcare system capacity constraints, which may also have been influenced by temporary factors such as the COVID-19 pandemic during 2020–2021 [27–29].

Fourth, the current analyses relied on detailed PSM and additional adjustment for OCM within multivariable CRR models. Both methods were warranted due to baseline differences between patients with TTN ≤ 3 vs. > 3 months across all four racial/ethnic groups, in order to minimize the effects of potential bias or confounders. Within each race/ethnicity, all patients with TTN > 3 months were matched to patients with TTN ≤ 3 months in one-to-two ratio. After PSM, no significant residual differences remained between patients with TTN ≤ 3 vs. > 3 months. To the best of our knowledge, no previous study relied on such methodology.

Fifth, we examined the association of TTN > 3 months and CSM within separate analyses for each of the four racial/ethnic groups. After PSM, CSM rates at 120 months after nephrectomy were higher in patients with TTN > 3 months than in those with TTN ≤ 3 months among both Caucasians (18.7 vs 12.2%) and Hispanics (11.0 vs 7.0%). In multivariable CRR models addressing CSM and adjusting for OCM and multiple covariates, TTN > 3 months was independently associated with higher CSM in Caucasians (sHR 1.57, p < 0.001) and Hispanics (sHR 1.55, p = 0.046). These findings were supported by sensitivity analyses using alternative TTN cutoffs and were consistent with formal interaction testing showing estimates close to unity for Hispanics relative to Caucasians. Conversely, no CSM differences according to TTN > 3 vs. ≤ 3 months were observed in African Americans and APIs. Therefore, TTN > 3 months failed to achieve independent predictor status in multivariable CRR models addressing CSM in the latter two racial/ethnic groups. These observations were supported by sensitivity analyses using alternative TTN cutoffs and by formal interaction testing suggesting attenuation of the effect of TTN > 3 months on CSM in African Americans and APIs relative to Caucasians, but without reaching statistical significance.

Taken together, the current findings provide the first evaluation of racial/ethnic differences in TTN and their prognostic implications for CSM in localized RCC. The proportion of patients with TTN > 3 months increased over time in all four examined racial/ethnic groups. Importantly, race/ethnicity-specific differences regarding TTN > 3 months were observed in two distinct settings: its prevalence and its association with CSM. With respect to prevalence, Hispanics and African Americans exhibited approximately two-fold higher proportions of TTN > 3 months compared with Caucasians and APIs. With respect to the association between TTN > 3 months and CSM, a clinically meaningful and statistically significant increase in CSM was observed in Caucasians and Hispanics, but not in African Americans or APIs. Therefore, Hispanics warrant particular attention, as they experienced both higher prevalence of TTN > 3 months and an adverse prognostic association between TTN > 3 months and CSM in subsequent competing-risks survival analyses. In Caucasians, TTN > 3 months also achieved independent predictor status for higher CSM in CRR models. Conversely, African American race/ethnicity was only associated with higher prevalence of TTN > 3 months, but TTN > 3 months did not independently predict higher CSM in those patients. Finally, neither of the two tested relationships was statistically significant in APIs.

Several explanations may account for the observed heterogeneity across racial/ethnic groups. With respect to differences in the prevalence of prolonged TTN, unmeasured access-to-care factors, referral patterns, and healthcare system-level variables not captured within SEER may contribute [30, 31]. With respect to the association between TTN and CSM, differential competing mortality may attenuate associations in some subgroups [32]. In addition, potential biological differences or variation in tumor behavior across racial/ethnic groups cannot be excluded [33]. Moreover, despite relying on a large population-based dataset, limited CSM event counts (Supplementary Table 6) in African American and API subgroups may have reduced statistical power to detect associations.

Additional limitations warrant consideration. First, our study shares the inherent constraints of retrospective analyses based on the SEER database, including the potential for misclassification. Second, we primarily focused on a TTN cutoff of > 3 months. Although additional sensitivity analyses using alternative cutoffs of > 2 and > 4 months were performed and yielded consistent results, an assessment of more markedly prolonged delay intervals (e.g., > 6 months) was not feasible due to limited patient numbers. The > 3 months threshold has been applied repeatedly in prior RCC investigations and, importantly, the proportion of patients exceeding this threshold represented a tangible and clinically meaningful target for TTN optimization across all four racial/ethnic groups [3–6]. Third, SEER does not provide information on the underlying reasons for prolonged TTN. It is possible that patient preference or preoperative medical optimization accounted for some. Fourth, because SEER reflects the structure and access patterns of the United States healthcare system, the observed racial/ethnic disparities in TTN and CSM may not be directly generalizable to healthcare systems in other countries, where insurance coverage, referral pathways, and treatment access differ substantially. Moreover, SEER lacks detailed healthcare system-level variables, such as facility type or hospital volume/bedsize. Fifth, data on comorbidity burden, surgical approach, tumor complexity and tumor growth kinetics are not captured in SEER, which may further refine interpretation of delay-related effects. Finally, SEER does not provide information on other oncologic control measures such as disease-free, progression-free, or metastasis-free survival, which precluded a more granular assessment of cancer control beyond CSM.

## Conclusions

In localized RCC patients treated with nephrectomy, TTN > 3 months became more prevalent over time in all four examined racial/ethnic groups. In Hispanics and African Americans, TTN > 3 months proportions were higher than in Caucasians and APIs. TTN > 3 months was independently associated with higher CSM only in Caucasians and Hispanics, but not in African Americans and APIs. Based on these observations, effect estimates derived from predominantly Caucasian cohorts should not be considered unbiased, as effects may differ by race/ethnicity. Therefore, stratified analyses or interaction testing are warranted in future studies.

## Supplementary Information

Below is the link to the electronic supplementary material.

Supplementary file1 (PDF 338 KB)

## Data Availability

Access to SEER data is available upon request through the SEER*Stat software after registration and approval by the National Cancer Institute. The authors had authorized access to these data. The data are not publicly available due to privacy and confidentiality restrictions imposed by the SEER program.
