# Supplementary material for: Racial/ethnic differences in time to nephrectomy and its association with cancer-specific mortality in localized renal cell carcinoma
Source: Int Urol Nephrol. 2026 Apr 16;58(9):3631–41. doi: 10.1007/s11255-026-05141-0 (PMC13506650; doi:10.1007/s11255-026-05141-0)
Supplement: Supplementary file 1 [file 11255_2026_5141_MOESM1_ESM.pdf]

Supplementary Figure 1: Love plot illustrating covariate balances before (unmatched, blue) and after (matched, red) separate propensity score matchings in Caucasians (a), Hispanics (b), African Americans (c), and Asians or Pacific Islanders (d).

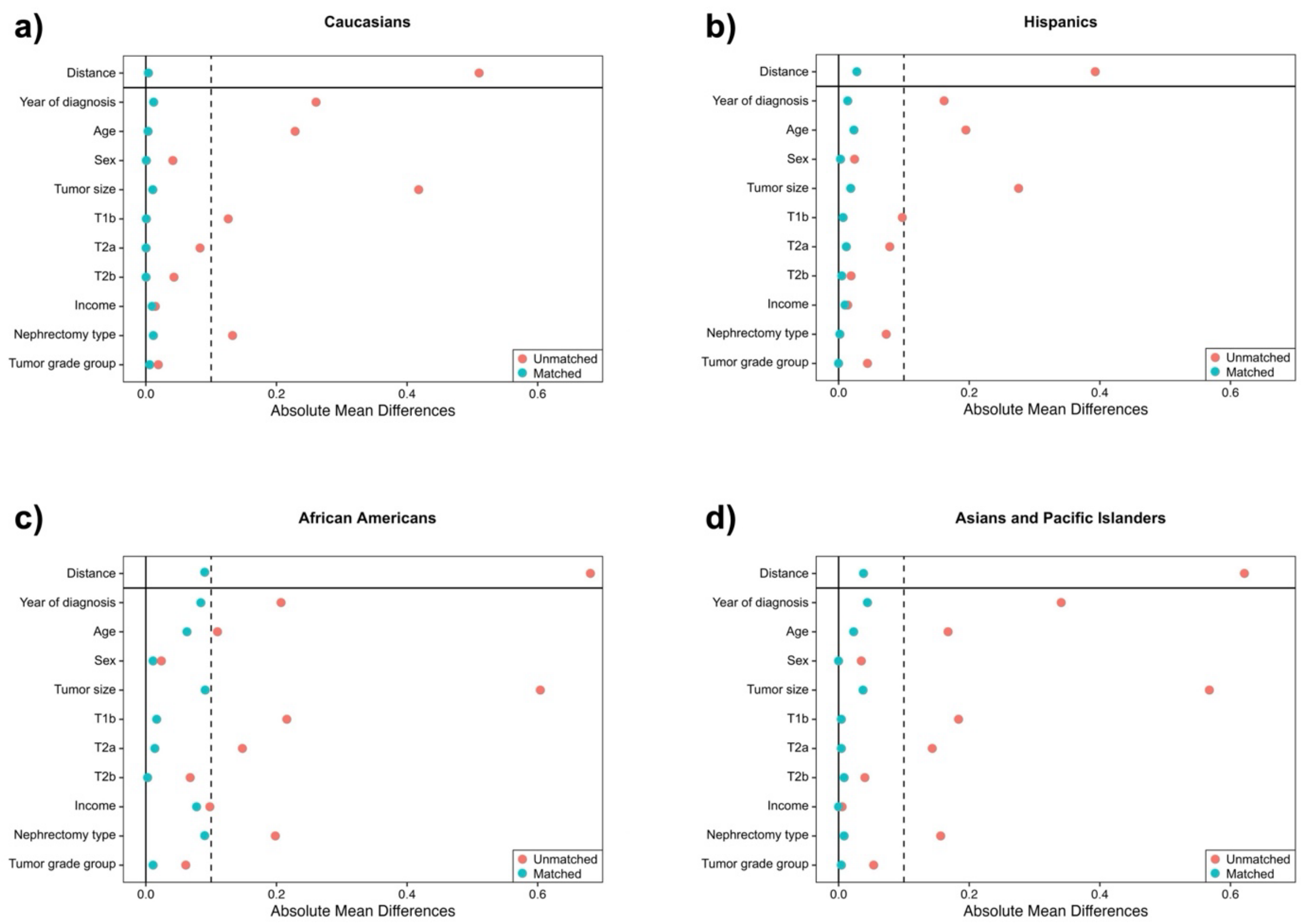

Supplementary Table 1: Multivariable logistic regression model (mLRM) predicting time to nephrectomy (TTN) >2 vs. ≤2 months according to race/ethnicity in 11,058 T1b–2 N0 M0 clear-cell renal cell carcinoma patients treated with partial or radical nephrectomy within the SEER database (2010–2021).

**Race/ethnicity**	**TTN ≤2 months**, n (%)	**TTN >2 months**, n (%)	**mLRM predicting TTN >2 months (vs. ≤2 months)**[a]		
			**Odds ratio**	**95% confidence interval**	**p-value**
**Caucasians**	5,182 (69.0%)	2,324 (31.0%)	Ref.		
**Hispanics**	1,194 (55.8%)	944 (44.2%)	1.95	1.76–2.16	**<0.001**
**African Americans**	376 (55.6%)	300 (44.4%)	1.93	1.64–2.28	**<0.001**
**Asians and Pacific Islanders**	502 (68.0%)	236 (32.0%)	1.10	0.92–1.30	0.3

[a] adjusted for year of diagnosis, age, sex, income, area of residence, tumor size, and nephrectomy type.

Supplementary Table 2: Multivariable logistic regression model (mLRM) predicting time to nephrectomy (TTN) >4 vs. ≤4 months according to race/ethnicity in 11,058 T1b–2 N0 M0 clear-cell renal cell carcinoma patients treated with partial or radical nephrectomy within the SEER database (2010–2021).

Race/ethnicity	TTN ≤4 months, n (%)	TTN >4 months, n (%)	mLRM predicting TTN >4 months (vs. ≤4 months)[a]		
			**Odds ratio**	**95% confidence interval**	**p-value**
**Caucasians**	6,841 (91.1%)	665 (8.9%)	Ref.		
**Hispanics**	1,811 (84.7%)	327 (15.3%)	1.97	1.70–2.28	**<0.001**
**African Americans**	565 (83.6%)	111 (16.4%)	2.09	1.64–2.61	**<0.001**
**Asians and Pacific Islanders**	669 (90.7%)	69 (9.3%)	1.13	0.86–1.47	0.4

[a] adjusted for year of diagnosis, age, sex, income, area of residence, tumor size, and nephrectomy type.

Supplementary Table 3: Multivariable competing risks regression models testing the association between time to nephrectomy (TTN) >2 vs. ≤2 months and cancer-specific mortality (CSM) according to race/ethnicity in T1b–2 N0 M0 clear-cell renal cell carcinoma patients treated with partial or radical nephrectomy within the SEER database (2010–2021) after propensity score matching.

Race/ethnicity	120-month CSM with TTN >2 months	120-month CSM with TTN ≤2 months	CRR addressing CSM of TTN >2 months (vs. ≤2 months)[a]		
			**sHR**	**95% confidence interval**	**p-value**
**Caucasians**	15.3%	12.6%	1.31	1.10–1.56	**0.003**
**Hispanics**	10.9%	8.3%	1.34	0.92–1.96	0.1
**African Americans**	12.0%	16.7%	0.74	0.42–1.32	0.3
**Asians and Pacific Islanders**	11.8%	9.8%	1.13	0.61–2.08	0.7

[a] adjusted for year of diagnosis, age, sex, income, area of residence, tumor size, nephrectomy type, and tumor grade group.

Supplementary Table 4: Multivariable competing risks regression models testing the association between time to nephrectomy (TTN) >4 vs. ≤4 months and cancer-specific mortality (CSM) according to race/ethnicity in T1b–2 N0 M0 clear-cell renal cell carcinoma patients treated with partial or radical nephrectomy within the SEER database (2010–2021) after propensity score matching.

Race/ethnicity	120-month CSM with TTN >4 months	120-month CSM with TTN ≤4 months	CRR addressing CSM of TTN >4 months (vs. ≤4 months)[a]		
			**sHR**	**95% confidence interval**	**p-value**
**Caucasians**	21.2%	11.1%	1.76	1.29–2.40	**<0.001**
**Hispanics**	11.9%	8.4%	1.59	1.05–2.46	**0.036**
**African Americans**	11.6%	11.1%	1.12	0.54–3.22	0.6
**Asians and Pacific Islanders**	11.4%	9.5%	0.89	0.22–2.91	0.7

[a] adjusted for year of diagnosis, age, sex, income, area of residence, tumor size, nephrectomy type, and tumor grade group.

Supplementary Table 5: Multivariable competing risks regression model including an interaction term between time to nephrectomy (TTN) >3 vs. ≤3 months and race/ethnicity addressing cancer-specific mortality (CSM) in T1b–2 N0 M0 clear-cell renal cell carcinoma patients treated with partial or radical nephrectomy within the SEER database (2010–2021) after propensity score matching.

Variable	CRR addressing CSM[a]		
	**sHR**	**95% confidence interval**	**p-value**
**TTN >3 months** (Ref. ≤3 months)	1.44	1.13–1.83	**0.004**
**Race/ethnicity** (Ref. Caucasians)			
Hispanics	0.71	0.52–0.98	**0.038**
African Americans	1.08	0.72–1.61	0.7
Asians and Pacific Islanders	1.36	0.86–2.16	0.2
**Interactions**			
TTN >3 months x Hispanics	0.96	0.57–1.61	0.9
TTN >3 months x African Americans	0.61	0.28–1.34	0.2
TTN >3 months x Asians and Pacific Islanders	0.55	0.23–1.30	0.2

[a] adjusted for year of diagnosis, age, sex, income, area of residence, tumor size, nephrectomy type, and tumor grade group.

Supplementary Table 6: Event counts for cancer-specific mortality (CSM) according to race/ethnicity in T1b–2 N0 M0 clear-cell renal cell carcinoma patients treated with partial or radical nephrectomy within the SEER database (2010–2021) before and after propensity score matching (PSM).

Race/ethnicity	CRR event counts	
	**Before PSM**	**After PSM**
**Caucasians**	645	271
**Hispanics**	119	76
**African Americans**	59	39
**Asians and Pacific Islanders**	51	22
